# Psychological Profile in Coronary Artery By-Pass Graft Patients vs. Valve Replacement Patients Entering Cardiac Rehabilitation after Surgery

**DOI:** 10.1038/s41598-018-32696-5

**Published:** 2018-09-26

**Authors:** Maddalena Modica, Paolo Castiglioni, Anna Minotti, Andrea Faini, Vittorio Racca, Maurizio Ferratini

**Affiliations:** 1 IRCCS Fondazione Don Carlo Gnocchi, Cardiology Rehabilitation Department, Milan, Italy; 2 IRCCS Fondazione Don Carlo Gnocchi, Milan, Italy; 3 Istituto Auxologico Italiano, IRCCS, Department of Cardiovascular Neural and Metabolic Sciences, Milan, Italy

## Abstract

Anxiety and depression are thought to influence the genesis of ischemic diseases and not of valvular diseases, but little is known on the psychological profile of cardiac patients after surgery. Aim of this study was to investigate differences in disease experience and mood between patients undergoing cardiac rehabilitation after coronary artery by-pass graft (CABG) or after valve replacement (VR). We studied 1,179 CABG and 737 VR patients who completed the Illness Behaviour Questionnaire and the Hospital Anxiety and Depression Scale after surgery. We tested the independent effect of the type of surgery by multivariate analysis and between-group differences in prevalence of clinically relevant scores. Relevant scores in the psychosomatic concern scale were more frequent in CABG than in VR patients. After correction by age, sex, education and marital status, scores of disease conviction and psychosomatic concern were higher in CABG patients, scores of denial were higher in VR patients. Unexpectedly, anxiety and depression scores did not differ between groups. Results suggest providing psychological support for anxiety and depression to both VR and CABG patients during cardiac rehabilitation, and planning differentiated interventions of cardiac rehabilitation and secondary prevention tailored to the specific psychological reactions of CABG and VR patients.

## Introduction

Cardiac rehabilitation considers an individual within a system in which biomedical, psychological and social variables interact, contributing to the genesis and evolution of the disease^1,2^. Therefore, rehabilitation programs after cardiac surgery should also involve multidisciplinary interventions aimed at changing the patients’ lifestyle and at preventing or reducing the negative psychosocial effects of the disease. The psychological profile of the patient may play a major role during cardiac rehabilitation, conditioning the adherence to the rehabilitation program and to the measures of secondary prevention necessary to restore health and quality of life^3^.

The psychological profile after cardiac surgery may depend on the preoperative clinical condition, on duration and severity of the disease and its symptoms, and on the type of cardiac surgery. In this context, numerous studies underlined the impact of anxiety and depression on the genesis of the ischemic heart disease. Since psychological factors are not considered to influence significantly the pathogenesis of valvular diseases, the majority of psychological studies have concentrated on coronary disease^4–10^, on its post-surgical course^11–14^, and on the efficacy of its rehabilitation programs^12,13^, rather than on valvular diseases. However, no studies have so far evaluated whether the prevalence of anxiety and depression actually differs between patients who underwent coronary surgery and patients who underwent valvular surgery.

For these reasons, main aim of the present study is to test the hypothesis that cardiac patients after coronary surgery have greater levels of anxiety and depression than patients who underwent valvular surgery. Testing this hypothesis is important to plan psychological interventions better tailored on the patient, in order to improve adherence to secondary prevention advice and, consequently, clinical outcomes.

In addition to anxiety and depression, other psychological variables specifically related to the disease experience can influence the course of rehabilitation and the adherence to secondary prevention, but the literature on these factors is much more limited. Therefore, second aim of our study is to provide a detailed description of the differences in disease experience and mood associated with these two types of cardiac disease. This was done by evaluating a large population of cardiac patients (N = 2204) undergoing post-surgical rehabilitation after coronary or valvular diseases with standard validated questionnaires.

## Results

Two hundred and eighty-eight of the 2,204 enrolled patients were excluded from the analysis because they had both coronary and valvular disease, and it was not possible to identify an unique indication for surgery. The final sample therefore consisted of 1,916 patients who had a mean age of 65.2 ±12.4 years and were prevalently males (71%); 28% were single, and just under half of them had had a high-school or university degree (46%).

The primary surgical indication was coronary artery by-pass graft (CABG) in 1179 cases and valve replacement (VR) surgery in 737 cases. Table 1 shows the general characteristics of the two groups, which were similar except for the percentage of males, higher in the CABG group, and of single patients, higher in the VR group. The questionnaires were administered a mean 20 days after surgery, with no significant difference between groups in the time from surgery (Table 1).

**Table 1:** General characteristics of patients by type of cardiac surgery.

	VR (n = 737)	CABG (n = 1179)
*Males (%)*	55.9%	80.0%^**^
*Age (yrs)*	65.8 (13)	66.1 (10.8)
*Time from surgery (days)*	17.8 (7.5)	22.5 (15.5)
*Education Level*		
*Low*	54.3%	53.8%
*High*	45.7%	46.2%
*Single (%)*	31.1%	25.4%^**^

Percentages or mean values (SD); the symbols °, * and ** indicate differences between groups significant at p < 0.10, p < 0.05 and p < 0.01 respectively, with p after unpaired *t* test for age, after Mann-Whitney U test for time from surgery, after chi-squared test for education level and single status; VR = Valve Replacement; CABG = Coronary Artery Bypass Graft.

### Anxiety and Depression

**Figure 1:**
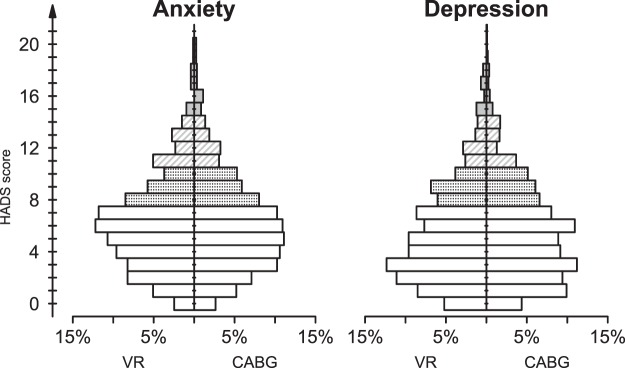
Distribution of HADS scores in VR and CABG patients. Open bars = “normal” scores; dotted bars = “mild” scores; dashed bars = “moderate” scores; grey bars = “severe” scores.

The Hospital Anxiety and Depression Scale (HADS) test detected levels of anxiety greater than normal in 31.8% of the patients, and depression levels greater than normal in 27.8% of the patients. Moreover, 13.1% of them had clinically relevant anxiety scores and 10.5% had clinically relevant depression scores. However, comparing CABG and VR groups, we found similar distributions of anxiety and depression scores (Fig. 1). Multivariate regression analysis assessed the effects of the type of surgery removing the possible influence of different confounders: age, sex, marital status and education level (Table 2). However, the multivariate analysis confirmed that HADS scores do not directly depend on the type of surgery: in fact, although anxiety and depression scores were higher in females, decreased with the educational level and depended on age, they did not depend significantly on surgery (we only found a tendency for higher depression scores in CABG patients which, however, did not reach the 5% significance level). Furthermore, the prevalence of HADS scores greater than normal or clinically relevant did not differ between VR and CABG patients (Table 3).

**Table 2:** Beta coefficients (standard error) after multivariate analysis for predicting HADS or IBQ scores separately.

	Cardiac Surgery (CABG = 0; VR = 1)	Age	Sex (F = 0; M = 1)	Education (0 = Low; 1 = High)	Single Status (No = 0; Yes = 1)
**HADS Scores**					
*Anxiety*	−0.03 (0.02)	−0.07 (0.02)^**^	−0.11 (0.02)^**^	−0.08 (0.02)^**^	−0.01 (0.02)
*Depression*	−0.04 (0.02)°	0.10 (0.02)^**^	−0.09 (0.02)^**^	−0.08 (0.02)^**^	0.03 (0.02)
**IBQ Scores**					
*Affective inhibition*	−0.02 (0.02)	0.02 (0.02)	0.03 (0.02)	−0.03 (0.02)	0.00 (0.02)
*Denial*	0.10 (0.02)^**^	0.11 (0.02)^**^	0.13 (0.02)^**^	−0.04 (0.02)	−0.10 (0.02)^**^
*Disease conviction*	−0.06 (0.02)^*^	0.02 (0.02)	−0.11 (0.02)^**^	−0.03 (0.02)	0.03 (0.02)
*Dysphoria*	−0.03 (0.02)	0.00 (0.02)	−0.14 (0.02)^**^	−0.04 (0.02)°	−0.02 (0.02)
*Hypochondriasis*	−0.01 (0.02)	0.00 (0.02)	−0.03 (0.02)	−0.12 (0.02)^**^	0.01 (0.02)
*Irritability*	−0.02 (0.02)	−0.04 (0.02)°	−0.01 (0.02)	0.02 (0.02)	−0.08 (0.02)^**^
*Psychosomatic concern*	−0.09 (0.02)^**^	−0.16 (0.02)^**^	−0.01 (0.02)	0.09 (0.02)^**^	0.02 (0.02)

The symbols °, * and ** indicate coefficients significant at p < 0.10, p < 0.05 and p < 0.01 respectively.

**Table 3:** Prevalence of HADS scores greater than normal or clinically relevant, and of clinically relevant IBQ scores, in VR vs. CABG groups, by sex.

	All		Males		Females	
	VR	CABG	VR	CABG	VR	CABG
**HADS Anxiety Score**						
*Greater than normal*	31.7%	31.9%	27.1%	29.7%	37.6%	41.0%
*Clinically relevant*	13.7%	12.7%	11.0%	11.8%	17.2%	16.2%
**HADS Depression Score**						
*Greater than normal*	27.3%	28.2%	23.4%	26.7%	32.3%	34.2%
*Clinically relevant*	10.6%	10.4%	7.8%	9.2%	14.1%	15.4%
**Clinically Relevant IBQ Scores**						
*Affective inhibition*	5.6%	4.9%	3.6%	5.3%	8.0%	3.4%*
*Denial*	29.6%	26.6%	31.9%	28.2%	26.6%	20.3% °
*Disease conviction*	7.7%	8.1%	5.3%	7.3%	10.8%	11.0%
*Dysphoria*	5.6%	5.2%	3.9%	4.8%	7.7%	6.8%
*Hypochondriasis*	4.6%	4.2%	4.8%	3.8%	4.3%	5.9%
*Irritability*	8.5%	7.8%	9.2%	8.1%	7.7%	6.8%
*Psychosomatic concern*	14.4%	20.3%^**^	15.5%	19.7% °	13.0%	22.5%^**^

The symbols °, * and ** indicate differences between groups significant at p < 0.10, p < 0.05 and p < 0.01 respectively; p after chi-squared test.

### Illness Behavior

**Figure 2:**
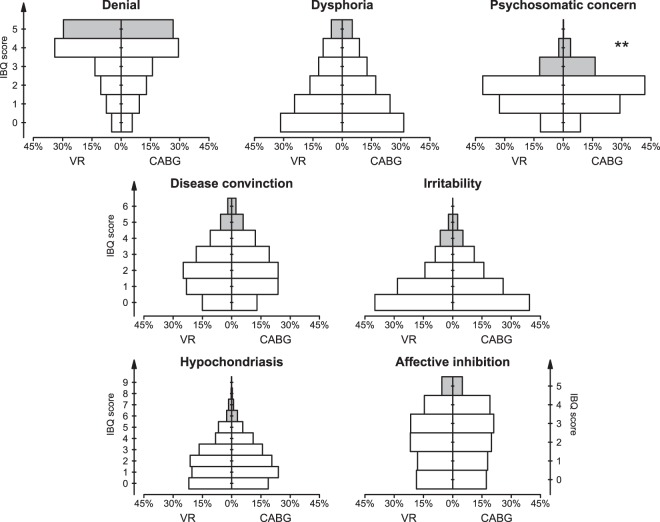
Distribution of IBQ scores by type of surgery. The solid grey bars indicate clinically relevant scores; the ** shows a statistically significant difference between groups in the prevalence of clinical relevant scores, at p < 0.01.

Over the whole group, the prevalence of clinically relevant IBQ scores was 5.2% for affective inhibition, 27.8% for denial, 7.9% for disease conviction, 5.3% for dysphoria, 4.4% for hypochondriasis, 8.1% for irritability and 18.0% for psychosomatic concern. Figure 2 and Table 3 show distributions of IBQ scores and prevalence of clinically relevant scores by type of surgery. The prevalence of clinically relevant scores in the Psychosomatic Concern scale was greater in CABG patients and considering the two sexes separately, the difference was more marked among females (Table 3). Furthermore, there was a difference in the prevalence of clinically relevant scores of the affective inhibition scale only among the female patients, which was more than twice as high among the women in the VR group (8.0% *vs*. 3.4%, Table 3).

Multivariate regression analysis removed the possible influence of the confounders age, sex, marital status and education level, and confirmed the higher psychosomatic concern scores in CABG patients. Furthermore, multivariate analysis also revealed that CABG surgery is associated with higher scores of disease conviction compared to VR surgery. The difference was not significant by directly comparing the two groups (Fig. 2) likely because of the higher prevalence of males in CABG patients and by the tendency of males to score lower than females in this IBQ scale. Multivariate analysis also revealed higher denial scores associated with VR surgery. In this case the difference was not significant directly comparing the two groups because of the greater prevalence of males in CABG patients and of singles in VR patients (Table 1), and because multivariate analysis points out that males denial more than females and that single patients denial less than not-single patients (Table 2).

## Discussion

Previous studies of patients with coronary disease have described the presence of anxiety and depression before and after the acute event^4–10,15^. What is new about our study, that focused the analysis on the rehabilitative period after surgery, is that it directly compared patients with coronary disease and those with valvular disease and, in addition to anxiety and depression, it also considered a broad spectrum of psychological attitudes that describe the disease experience in a more detailed and complete manner.

### Anxiety and depression

Anxiety and depression can greatly influence the onset and course of cardiac disease. Epidemiological studies have shown that the prevalence of major depression is three times higher among ischemic subjects than in the general population^16^, and that depression favors the onset of ischemic disease and worsens its course in patients who already have it^17^. Depression favors heart disease by means of physiopathological mechanisms such as serotonin metabolism, sympathetic hypertonia, and lipid metabolism^18^, and behavioral mechanisms that induce unhealthy lifestyles such as poor treatment compliance, an inappropriate diet, sedentariness, smoking and substance abuse.

Previous studies have also found that anxiety is frequent in subjects with chronic coronary heart disease^19^ and in patients undergoing rehabilitation after a coronary event^20^. It can hinder psychosocial adjustment and cardiovascular recovery, and worsen the quality of life^21^. Anxious patients do not accept information concerning lifestyle modifications and, consequently, do not respect the medical prescriptions necessary for their rehabilitation^22^. Persistent anxiety is also predictive of some forms of incapacitating disabilities, of an increased number of physical symptoms, and of a poor functional status^23^.

In this regard, our study shows that about one-third of the patients beginning a post-surgical cardiac rehabilitation program suffer from anxiety and depression, and that more than one-tenth is afflicted by severe depression and clinically relevant anxiety. Published data indicate that the prevalence of anxiety and depression in cardiac patients varies widely, from 15% to 50%^24^, discrepancies that may be due to differences in the diagnostic instruments used. The HADS questionnaire we used does not consider the somatic aspects of anxiety and depression such as disordered sleep or appetite, or other frequent postoperative symptoms, which probably reduces over-diagnosis and the number of false positive results, and may explain the higher percentages reported in other studies. In any case, we showed that the prevalence of anxiety and depression was substantially similar in the two groups, an unexpected finding because anxiety and depression have been described as independent risk factors for coronary diseases but the genesis of valvular diseases is considered to be of biological, degenerative and organic origin (e.g., caused by rheumatic and/or congenital disease, by pathological calcium deposition, etc.) and therefore not conditioned by psychological factors.

The prevalence of anxiety or depression has not been reported to differ in cardiac patients after CABG surgery^25,26^. However, the period of hospital rehabilitation (which coincides with our period of clinical observation) may be characterized by the perception of risk and fears of postoperative complications and only limited functional recovery, giving rise to syndromes of anxiety and depression also in patients with valvular disease. In our study, no data are available concerning the patients’ preoperative psychopathological history, and therefore we do not know whether the CABG and VR groups differed in anxiety and depression before surgery and whether the stressful event of surgery may have canceled the differences between the two groups. In any case, since our two populations were similar in terms of postoperative anxiety and depression, our results underline the importance to ensure psychological support during the postoperative rehabilitation period not only to ischemic patients, but also to patients with valvular disease, to manage promptly anxious/depressive syndromes or any other problems that may emerge, and thus favor global recovery and the adoption of healthy behaviors.

To interpret our data correctly, it is necessary to remember that, in the general population, men are less frequently affected by anxiety and depression than women. This was also reflected in our patients (Tables 2 and 3), confirming previous findings on sex differences in the psychological profile of a generic population of cardiac patients^27^. Consequently, because of the different sex composition of our VR and CABG groups, with a marked prevalence of males in CABG patients (Table 1), gender differences may hide between-group trends more directly related to the nature of the disease. However, even performing the comparisons separately by sex, or considering sex as one of the confounders in multivariate analysis, our study did not find differences between groups.

### Illness behavior

The scores of the psychosomatic concern scale show that the CABG patients were inclined to attribute a psychological origin to the disease and its symptoms more than the VR patients. Increasing public awareness of the pathogenetic importance of coronary risk factors means that patients know of the role that stress-related factors play in the development of the disease. Another novel finding is that after adjustment for confounders, VR patients resulted to be inclined to deny the stressing aspects of their lives more than CABG patients (Table 2). One of the critical aspects of managing the emotional reactions triggered by a cardiac event is the difficulty that patients have in acknowledging their emotions, which may lead to them adopting dysfunctional adaptive mechanisms such as denial. It is reasonable to hypothesize that denial is a mechanism of adapting to valvular disease over time. Unlike coronary heart disease, which has an acute onset, valvular disease is generally characterized by a chronic history lasting months or years and this, together with the need to recover rapidly, could induce patients to adapt to the disease and underestimate its emotive component.

A further result emerged from the by-sex analysis of the clinically relevant scores of affective inhibition: women who tend not to express their difficulties or share them socially are more frequent in the VR group (Table 3). This was unexpected insofar as affective inhibition is a trait of type D personality, which is a risk factor for coronary heart disease^28,29^, and actually this appears reflected in the albeit non-significantly higher prevalence of clinically relevant scores among the men with coronary disease. Future studies should investigate whether this psychological factor contributes to limiting women’s access to rehabilitation treatment as it has been shown in other studies that their need to resume their family roles rapidly conflicts with the time required for postoperative care and therefore with their participation in rehabilitation programs.

Three limitations that could be subject of further investigation should be mentioned. First, it was not possible to administer psychological questionnaires before surgery, which may have allowed a better understanding of the high levels of anxiety and depression. However, this does not affect the study objective, which was to identify the problems that should guide psychological interventions during rehabilitation and which therefore requires psychological evaluations at the beginning of the rehabilitation program. Second, since the evaluation period was close to the stressful event of surgery, we cannot exclude that HADS and IBQ scores may be different after some weeks of in-patient cardiac rehabilitation. Third, there are no data available concerning postoperative complications, which may affect patients’ psychological experiences. However, as the psychological interviews were conducted a mean of 20 days after surgery without a significant difference between groups, it may be presumed that most of the patients had a postoperative course free from major complications.

In conclusions, syndromes of anxiety and depression substantially similar in VR and CABG patients means that also in VR patients it is important to diagnose anxiety and depression and adopt psychological support measures. On the other hand, there are differences in psychosomatic concern, denial and disease conviction due to the type of surgery and, in the case of females, differences between groups in clinically relevant scores of affective inhibition. These aspects might negatively affect behavior and treatment compliance during the rehabilitation phase. Structured psychological support should therefore be made available to all patients after cardiac surgery, and not only to those who have undergone CABG.

## Methods

### Subjects

All of the adult patients consecutively referred to our rehabilitation unit after cardiac surgery between 2005 and 2015, undergoing CABG or VR surgery, were considered eligible for the study. However, the patients who could not fully understand the questionnaires because they could not understand Italian sufficiently well or because of cognitive impairments (less than 4% of the referred population) were excluded, as were those participating in other research studies and those who received transcatheter aortic valve implantation. The enrolled population consisted of 2,204 cardiac patients of both sexes aged 21–95 years. Data relating to 1,323 patients have been previously used to evaluate gender differences in the psychological response to cardiac surgery^27^.

The study was carried out in accordance with the 1975 Declaration of Helsinki and was approved by the Ethics Committee of the Don C. Gnocchi Foundation, Milan, Italy. All of the patients gave their informed consent.

### Data collection

Within 2–3 days of admission, a psychologist interviewed each patient and recorded his/her age, level of education (dichotomously classified as “low”, if less than high school, and “high”, if high school or a higher degree), and marital status (single or not single). During the same interview, the patients were administered the Hospital Anxiety and Depression Scale (HADS)^30^ and the Illness Behaviour Questionnaire (IBQ)^31^, both adapted and validated for the Italian language^32,33^.

HADS consists of two series of seven items (one for anxiety and one for depression) that are scored 0–3, and so final scores range from 0 to 21. HADS scores exclude psychosomatic factors such as loss of appetite, insomnia or headache, which may be directly due to organic illness or treatments. Scores of anxiety and depression are categorized in 4 classes, defined as “normal” (scores between 0 and 7); “mild” (between 8 and 10); “moderate” (between 11 and 14) and “severe” (between 15 and 21). Accordingly, in our study scores greater than 7 indicate caseness, scores greater than 10 indicate a clinically relevant mood disorder.

The IBQ is a self-evaluation instrument that explores the attitudes, ideas and feelings of the patient relating to his/her disease. It consists of 62 items with binary yes/no answers, and its subscales quantify the extent to which illness influences seven psychological aspects: hypochondriasis (an anxious concern of patients about their own state of health); disease conviction (affirmation of psychosomatic diseases despite physician reassurances); psychosomatic concern (a tendency to attribute illness to psychological rather than somatic causes); affective inhibition (difficulty in expressing feelings, particularly negative feelings, to others); dysphoria (feelings of anxiety, sadness and indifference); denial (a tendency to deny any life stresses, attributing their own problems to somatic illnesses); and irritability (angry feelings and interpersonal frictions). Hypochondriasis is scored 0–9, irritability and disease conviction are scored 0–6, and the other subscales are scored 0–5. Clinically relevant scores are ≥3 for psychosomatic concern; ≥4 for irritability; ≥5 for disease conviction, affective inhibition, dysphoria, and denial; and ≥6 for hypochondriasis. This test has been previously applied in populations of cardiac patients to evaluate the impact of disease severity on perceived well-being^34,35^ and sex-related differences in mood disorders associated with the cardiac illness^27^.

The diagnosis, type of procedure, and the time since the procedure, were recorded as written in the medical records of each patient. Furthermore, the patients were assigned to the CABG or VR group on the basis of the primary indication for cardiac surgery.

### Statistics

The literature previously reported differences between male and female cardiac patients in the prevalence of anxiety and depression^27,36,37^ and in illness behavior^27^. For this reason, differences between VR and CABG groups were assessed not only on the whole population of patients, but also on male and female subgroups separately.

Differences between the VR and CABG groups in the prevalence of anxiety and depression or of clinically relevant HADS or IBQ scores were tested using Pearson’s chi-squared test. The chi-squared test was also used to compare the groups in terms of the prevalence of being single, male, and educated at the different levels, whereas age was compared by unpaired *t* test. Multivariate linear regression analysis was used to identify the independent predictors of each HADS or IBQ score separately. The predictors were the type of surgery (CABG = 0 and VR = 1), age, sex (female = 0, male = 1), education level (low = 0, high = 1) and single status (not single = 0, single = 1). The analyses were made with STATISTICA software package (StatSoft, Inc, Tulsa, OK).

## Data Availability

The datasets analyzed during the current study are available from the corresponding author on reasonable request.
